# Evidence-based management of eye care delivery

**Published:** 2018-07-31

**Authors:** B S Ganesh Babu, Thulasiraj D Ravilla

**Affiliations:** 1 Senior Manager: IT, Aravind Eye Care System; 2 Director of Operations: Aravind Eye Care System

**This article is the the first in series on evidence based eye care delivery. The series highlights the importance of using evidence for planning and effectively managing eye care – both at programme and hospital levels.**

**Figure:**
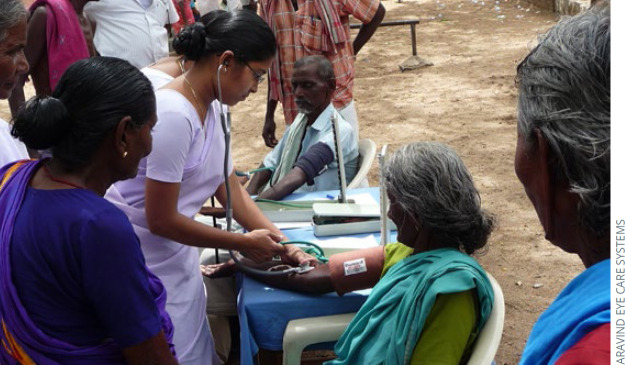
Productivity depends on right mix and number of allied ophthalmic personnel. INDIA

Evidence-based management (EBM) is essentially about consciously using sound information for effective management and decision-making.^1^ It is an approach that should be practiced to improve the way decisions are made in day–to-day work. This requires having the right evidence or information and a habit of taking decisions based on such evidence.

It is a well-established practice in medicine for many years and in the recent times getting popular in other fields.^2^ Akin to physicians, evidence is important for managers looking to ‘cure’ their organisation's ills. Just as it is untenable for doctors to treat patients without evidence from patient history and appropriate investigations, it is equally or more dangerous when strategic or operational decisions are taken without appropriate evidence.^1^ In this context, it is important to understand what should be measured and monitored to efficiently manage hospitals and healthcare programmes.

In the application of evidence, it is critical that we identify the right metrics so that it serves the organisational goals and brings in excellence in the operations. In this context, the following framework could be helpful. Let us consider the following situation to bring in a practical understanding of this.

Primary eye care or vision centres (VC) are seen as a viable strategy to ensure universal coverage for eye care. This would require everyone in need of eye care accessing the VC and receiving appropriate treatment or referral to address all eye conditions at a cost that is affordable. This requires, amongst other things that the VC has adequate demand. We can explore, how evidence can help the primary eye care strategy to succeed.

## Framework for defining metrics

Right metrics are those that help us figure out ‘the right things to do’ and then ensure that we ‘do them right’. The metrics to manage programmes or projects at strategic level are usually defined at the planning stage itself using Log Frame, a management technique that summarises a project into a 4×4 table, based on goals, objectives and specific tasks of the project, thus ensuring that all aspects are comprehensively covered. Similarly the following framework will ensure comprehensiveness of the metrics identified, to manage eye care effectively.

**Purpose:** Metrics to assess whether we are aligned with the vision and mission, as well as achieving it. For a VC, the possible metrics could be

- % of population reached out of the service area population
- % of refractive error patients examined against estimated annual need
- % of cataract surgeries against annual need
- % of glaucoma cases identified against estimated need in service population

**Demand:** Metrics to know where the patients come from and where they don't, the variations; health seeking behaviour viz. how early they come; conditions they come for

- Village level distribution of patients
- Age/gender distribution of patients
- Vision or average duration of eye problem– how early they come
- Diagnosis distribution – for what conditions they come
- Purpose of visit – in addition to eye care needs, patients could also be coming for replenishment of medicines, blood sugar monitoring or information.

**Compliance:** Metrics to continually know what proportion comply with the advised treatment or follow-up;

- % patients buy/use spectacle as per prescription
- % patients reporting to base hospital as per referral
- % patients buy/use medicine as per prescription
- % patients underwent surgery as per advise

**Quality:** Metrics to assess care in terms of surgical outcomes as well as patient satisfaction with service and how it impacts demand.

- % of patients needed referral as per base hospital
- % of patients returned with complaints after using spectacles
- % of patients with postoperative visual outcome conforming to WHO standard^3^
- % of patients who rate the overall experience as excellent
- Net promoter score - measures willingness of a patient to recommend to others

**Human resource:** Metrics to ensure that right number and mix of staff are available; productivity measures, retention and attrition rates; employee satisfaction and engagement

- Number of patients handled per day
- Average time-taken per patient
- Punctuality in opening the centre
- Number of days a VC centre had to be closed due to lack of human resources

**Finance:** Metrics to ensure financial viability; awareness of trends in expenses and revenues

- % of total income over expenses - cost recovery
- Costs incurred per patient

**External:** Metrics to monitor activities beyond our direct control but would affect our organisation.

- Are there any new/other eye care providers started serving
- Population movement (any migration happening in service region)

## Effective use of evidence

Sometimes data by itself may inform the status. However to trigger actions for improvement, making comparisons against a benchmark can be effective. Such benchmarks can be defined based on community needs, targets, external performance and previous achievements. For instance, if we assume that 20% of the service area population would need eye care services then this can become the target for coverage; compliance target could be set at 80% for patients referred to hospital and to ensure financial viability, the costs recovery through patient revenues could be set as more than 100%. When there are multiple centres, comparison of the same metrics across centres facilitates cross learning and improvements. Without having such standards as benchmarks to compare, the evidence on hand cannot translate to corrective measures.

## Information is needed at all levels

We need to recognise that information is required at all levels and the design of the information system should reflect it. The senior leadership would be interested in strategic information to know the performance of the primary eye care centre at macro level in terms of population coverage, patient load, revenue, etc. While a mid-level manager would be interested to know about the compliance rate of referrals, surgery, follow-up exam, etc. He or she would need this periodically as well as a comparison across all the centres, for providing appropriate support. Whereas the technician delivering patient care and managing daily operations, would like to review details such as patients who are due for a visit or those who are non-compliant to surgery, so that he or she can act on it.

## Practicing evidence-based management

**Figure 1:**
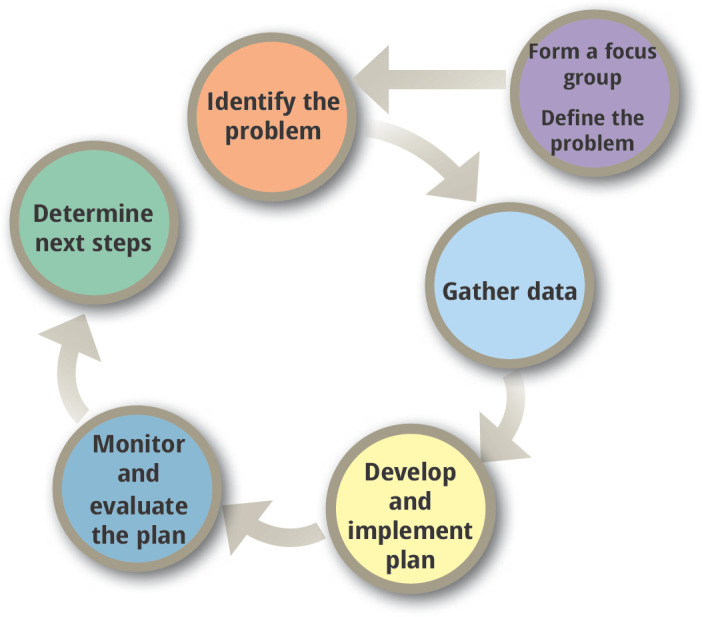
Problem solving process

Practicing EBM requires an organisational and individual discipline of making decisions based on evidence. When top management demonstrates this behaviour, others in the organisation will follow. Information generated should be reviewed periodically at appropriate levels. Such review will invariably throw up action items and these need to be followed through (see Figure 1). These practices ensure continuous improvement and a healthy work environment.

## Practical considerations for generating good evidence

Data quality or its timely availability is often the reason for not using evidence. The root cause of poor quality of data could be due to poor design of the system, lack of training or not having the required technology. However we should recognise that the quality of the data depends on how effectively we use them. Regular usage of information is what improves the quality of data and its timely availability.

The system should be designed to capture data as part of the workflow and allow the users to generate daily report to reflect the quality of the data captured. It is important to verify the quality of data as they are generated and at the end of each day, to avoid surprises when we generate periodic reports.

## Role of technology in practicing EBM

Information technology (IT) plays a significant role in building an information system. It is becoming increasingly affordable and easy to use. It is now possible to give information in real-time which can be accessed from anywhere at any time.

## Conclusion

Practicing EBM requires access to accurate and current information as needed; trigger exceptions; highlight areas needing focus, etc. All of this is possible with IT enabled systems. There are many tools available including open-source products such as spreadsheets, databases, statistical packages and Power BI that could help to build a process to capture, process and share the information more efficiently. This in turn facilitates action on the basis of the information, resulting in improved performance or outcomes.
